# Outcome after intubation for septic shock with respiratory distress and hemodynamic compromise: an observational study

**DOI:** 10.1186/s12871-021-01471-x

**Published:** 2021-10-25

**Authors:** Ting Yang, Yongchun Shen, John G. Park, Phillip J. Schulte, Andrew C. Hanson, Vitaly Herasevich, Yue Dong, Philippe R. Bauer

**Affiliations:** 1 Respiratory and Critical Care Medicine, West China Hospital, Sichuan University, Chengdu, 610041 Sichuan China; 2 Pulmonary and Critical Care Medicine, Mayo Clinic, Rochester, MN 55905 USA; 3 Health Science Research – Biomedical Statistics and Informatics, Mayo Clinic, Rochester, MN 55905 USA; 4 Critical Care Medicine, Mayo Clinic, Rochester, MN 55905 USA

**Keywords:** Septic shock, Respiratory failure, Endotracheal intubation, Outcome

## Abstract

**Background:**

Acute respiratory failure in septic patients contributes to higher in-hospital mortality. Intubation may improve outcome but there are no specific criteria for intubation. Intubation of septic patients with respiratory distress and hemodynamic compromise may result in clinical deterioration and precipitate cardiovascular failure. The decision to intubate is complex and multifactorial. The purpose of this study was to evaluate the impact of intubation in patients with respiratory distress and predominant hemodynamic instability within 24 h after ICU admission for septic shock.

**Methods:**

We conducted a retrospective analysis of a prospective registry of adult patients with septic shock admitted to the medical ICU at Mayo Clinic, between April 30, 2014 and December 31, 2017. Septic shock was defined by persistent lactate > 4 mmol/L, mean arterial pressure < 65 mmHg, or vasopressor use after 30 mL/kg fluid boluses and suspected or confirmed infection. Patients who remained hospitalized in the ICU at 24 h were separated into intubated while in the ICU and non-intubated groups. The primary outcome was hospital mortality. The first analysis used linear regression models and the second analysis used time-dependent propensity score matching to match intubated to non-intubated patients.

**Results:**

Overall, 358 (33%) ICU patients were eventually intubated after their ICU admission and 738 (67%) were not. Intubated patients were younger, transferred more often from an outside facility, more critically ill, had more lung infection, and achieved blood pressure goals more often, but lactate normalization within 6 h occurred less often. Among those who remained hospitalized in the ICU 24 h after sepsis diagnosis, the crude in-hospital mortality was higher in intubated than non-intubated patients, 89 (26%) vs. 82 (12%), *p* < 0.001, as was the ICU mortality and ICU and hospital length of stay. After adjustment, intubation showed no effect on hospital mortality but resulted in fewer hospital-free days through day 28. One-to-one propensity resulted in similar conclusion.

**Conclusions:**

Intubation within 24 h of sepsis was not associated with hospital mortality but resulted in fewer 28-day hospital-free days. Although intubation remains a high-risk procedure, we did not identify an increased risk in mortality among septic shock patients with predominant hemodynamic compromise.

**Supplementary Information:**

The online version contains supplementary material available at 10.1186/s12871-021-01471-x.

## Background

Septic shock remains common and is associated with high mortality [1–3]. Early recognition and management of septic shock with appropriate antibiotics, fluids, vasopressors, and source control is the cornerstone of treatment aimed at reducing morbidity and mortality [4, 5]. Sepsis-related acute respiratory failure is frequent, occurs early, requires non-invasive or invasive ventilator support, and may contribute to higher in-hospital mortality [6, 7]. Intubation and invasive mechanical ventilation are a common rescue procedure in the management of septic patients with acute respiratory failure. Although guidelines recommend a protecting lung strategy once mechanically ventilated, they do not provide any recommendation on the indication or the timing of intubation [8].

The decision to intubate a critically ill septic patient is complex and multifactorial. It does not rely solely on the severity or trajectory of the respiratory failure but may depend on various patient’s characteristics as well as provider preference and the strain on the healthcare system [9, 10]. By avoiding complications associated with delayed intubation, intubation within 24 h of ICU admission may improve outcomes. Deferring intubation in patients with acute respiratory distress and inappropriate reliance on non-invasive ventilation has been associated with increased mortality [11, 12]. In a large cohort of critically ill patients requiring invasive mechanical ventilation, intubation that was delayed by more than 2 days after admission was associated with higher in-hospital mortality [13]. Delaying intubation in patients with severe community-acquired pneumonia were also associated with worse outcomes in those who ultimately required invasive mechanical ventilation [14].

Intubation is often required in the most critically ill patient. It may, however, worsen cardio-circulatory failure after intubation, and premature intubation may expose patients to unnecessary risks [15, 16] and complications [17, 18]. In a survey of 186 intensivists from 30 countries on the criteria to initiate invasive ventilation in septic patients with respiratory distress, there was a large consensus (95%) that intubation should be performed in patients with predominant neurologic criteria (e.g. Glasgow Coma Scale less than 8, agitation, confusion) or respiratory criteria (cyanosis, tachypnea, high oxygen delivery, or clinical respiratory distress) [19]. There was much less consensus (76.1%) as a reason for intubation in the presence of hemodynamic criteria (lactic acidosis, hypotension, poor skin perfusion, or vasopressor use) and 51% of respondents believed that intubation and invasive mechanical ventilation would worsen patients with septic shock [19]. The decision to intubate a patient with sepsis, respiratory distress, and hemodynamic compromise is often hampered by the fear of worsening clinical condition and precipitating cardiovascular failure, like what has been reported with intubation in patients with salicylate overdose [20]. However, delaying intubation may have disastrous consequences.

Thus, the aim of this study was to evaluate the impact of intubation and mechanical ventilation in septic shock patients with respiratory distress and hemodynamic compromise, within 24 h after ICU admission for septic shock. We hypothesized that deferring intubation would be associated with worse in-hospital mortality and reduced hospital-free days in patients with septic shock.

## Methods

All methods were carried out in accordance with relevant guidelines and regulations. STROBE reporting guidelines for observational studies were followed [21].

### Patients

This study was approved by the Institutional Review Board of Mayo Clinic, Rochester, Minnesota, USA (#14–008754) who waived informed consent. We only reviewed the electronic medical records of patients who had given prior authorization to have their chart reviewed for research purpose. Every patient treated at our institution is required to indicate whether he/she authorizes his/her chart to be reviewed for research purpose. All consecutive patients with septic shock by sepsis 2–0 criteria [22], admitted to the 24-bed Medical Intensive Care Unit (ICU) of a tertiary medical center, were prospectively collected in a registry for an ongoing quality improvement project previously described [23]. Briefly, patients with septic shock were initially identified by screening criteria using an automated surveillance algorithm (sepsis “sniffer”) [24]. Quality coach nurses subsequently checked the chart of these patients to confirm the diagnosis before the data were manually entered in the database. Team monitors performed periodic checks to guarantee the validity of the data. Patients were included in the registry if they met the following criteria: (I) Age equal or greater than 18 years; (II) sepsis onset diagnosed upon ICU admission, defined by the presence of a clinically suspected or diagnosed infection in association with systemic inflammatory response criteria [22]; (III) if multiple ICU admissions occurred, only the first admission was recorded. The exclusion criteria included those with a do-not-resuscitate/do-not-intubate order within the first 48 h following ICU admission, patients intubated prior to ICU admission, and patients or legal authorized representative who declined research authorization.

From the registry, for a period spanning from April 30, 2014 (date of inception of the registry) to December 31, 2017 (time when this study was initiated), we reviewed retrospectively the electronic medical record of those adult patients admitted to the ICU with septic shock defined by persistent lactate level > 4 mmol/L, mean arterial pressure < 65 mmHg or vasopressor use after 30 mL/kg fluid boluses with a clinically diagnosed or suspected source of infection. During that period, a sepsis management bundle was embedded into the computerized physician order entry of the electronic medical record and included at least a 6-h follow up to comply with the best practice of the sepsis bundle. The ICU team also followed a procedural checklist for intubation with automatic back up from anesthesiology and the ventilator management followed a ventilator bundle adhering to a lung protective strategy with a high compliance that included low tidal volume (6 ml/kg of predicted body weight, range 4 to 8 ml/kg), while maintaining a plateau pressure at 30 cm H_2_O or below, most often by volume control mode and less often pressure control mode. The adhered to ventilator bundle included (unless contra-indicated) elevation of the head of the bed at 30 to 45 degrees, deep vein thrombosis prophylaxis, peptic ulcer prophylaxis, and topical chlorhexidine.

### Data collection

Patient characteristics were extracted from the ICU Data Mart, a Microsoft Structured Query language database, where all the static data, including the State death registry, are updated quarterly [25]. The extracted data included: Age, gender, admission source, Acute Physiology Score, Acute Physiology and Chronic Health Evaluation-III (APACHE-III), Sequential Organ Failure Assessment (SOFA) score, lactate level, basic metabolic panel, and the source and type of infection. Patients who remained in ICU at 24 h following sepsis onset were divided into two groups according to the need for intubation and invasive mechanical ventilation within 24 h.

### Statistical analysis

#### Outcome definitions

A statistical analysis plan was developed by the study team, drafted by biostatisticians on this manuscript and revised together, prior to statistical analysis of the data. The main outcome was hospital mortality. Secondary outcomes included ICU mortality, ICU- and 28-day hospital-free days. Specifically, a patient who died in the ICU or hospital would have zero ICU- or hospital-free days, respectively. The goal was to assess the association between intubation and outcomes; we report two different statistical approaches to this goal that are complementary but together provide added robustness to assumptions [26–29]. The first analysis uses linear regression models to compare those intubated in the first 24 h after sepsis onset to those not intubated in the first 24 h; the second analysis uses time-dependent propensity score matching to match intubated to non-intubated patients after ICU admission for comparison.

#### Unadjusted and adjusted logistic regression models

In the first analysis, we identified patients who remained hospitalized in the ICU at 24 h after sepsis onset and identified those who were intubated during that 24-h period (after excluding those who were intubated prior to admission). Continuous variables are summarized as median (interquartile range) and compared between patients intubated and patients not intubated using rank-sum tests. Categorical variables are summarized as frequencies and percentages and compared using Chi-squared tests. ICU and hospital length of stay are summarized only for patients who were discharged alive from the ICU and hospital respectively. The association between intubation and hospital mortality was assessed using unadjusted and adjusted logistic regression models. ICU mortality was analyzed similarly. The association between intubation and hospital-free days defined within 28 days was analyzed using unadjusted and adjusted linear regression models. Hospital-free days were defined as 28 minus length of stay but with subjects who died having 0 hospital-free days. Length of stay (among those discharged alive) was calculated using date-time of discharge minus date-time of ICU admission [30]. This approach is preferred to analysis of length of stay so that mortality is defined as the worst outcome response and larger response equates to discharge alive with shorter length of stay. ICU-free days were analyzed similarly using ICU discharge date-time. Adjustment variables included age, sex, ICU admission source, APACHE III and SOFA score on ICU day 1, resolution of hypotension (3 or more consecutive measurements of mean arterial pressure > 65 mmHg) within 6 h, resolution (decrease by 50% or normalization) of lactic acidosis within 6 h, and use of non-invasive ventilation within 24 h after the onset of septic shock. Cumulative incidence of intubation in the 24 h following septic shock and cumulative incidence of hospital discharge according to intubation status at 24 h following septic shock are presented.

#### Time-dependent propensity score matching

In the second analysis, we used time-dependent propensity score matching to match intubated patients with other patients who were not intubated. Four discrete time-periods were used (0–6 h, 7–12 h, 13–18 h, and 19–24 h after ICU admission) to facilitate data collection and imputation of missing data. For a patient intubated in the time interval after admission, we identified all subjects who were alive and not intubated at the end of the time interval as potential untreated matches. The propensity to be intubated was estimated using time-dependent Cox proportional hazards models over the 4-time-period intervals. The probability of intubation at or before the end of each interval was obtained as 1 minus the survival estimate from the Cox model using the Breslow estimator. Variables used in the propensity score calculation included time-independent variables: age, sex, source of admission, pre-ICU hospital length of stay, and year of admission; as well as time-dependent variables: acute physiology score (APS) and laboratory values (anion gap, bicarbonate, hematocrit, potassium, creatinine, glucose, sodium, blood urea nitrogen, bilirubin, pH, and lactate). Laboratory values were the most recent prior to intubation among intubated patients and last observed in each interval for those not intubated; APS was similarly updated in a time-dependent manner using worst observed labs in the prior 6 h (6 h prior to intubation among intubated patients or 6 h prior to end of interval among non-intubated patients). Functional form and interactions were assessed in the propensity score model; restricted cubic splines were used where appropriate for non-linear functional forms and a sex by admission source interaction was included.

In each period, we matched one-to-one, with replacement, intubated to non-intubated patients using the time-dependent propensity score. Patients intubated later (for example, between 19 and 24 h) could serve as non-intubated matches for patients intubated in the earlier intervals. Balance characteristics are described before and after matching using absolute standardized differences. Mortality and hospital-free days were analyzed in the matched sample using logistic or linear regression, respectively, with generalized estimating equations robust variance estimates to account for matching with replacement. Multiple imputations using the fully conditional specification approach were used for missing data assuming the missing at random mechanism [31, 32]. Of the 29 imputed variables, some of the variables were missing with different frequencies but 12 of the variables were missing < 10% of the time. The 5 variables with the most missing values were bilirubin (79% missing), pH (69%), platelets (55%), white blood cells (51%), and hematocrit (49%). Twenty imputed datasets were created, and analyses reflect the combined estimate accounting for variation due to missing data. In the propensity-matched analysis, standardized differences are described for the first imputed dataset.

Data were analyzed using SAS 9.4 (SAS Institute, Cary, NC, USA).

## Results

### Demographics and clinical data

**Fig. 1:**
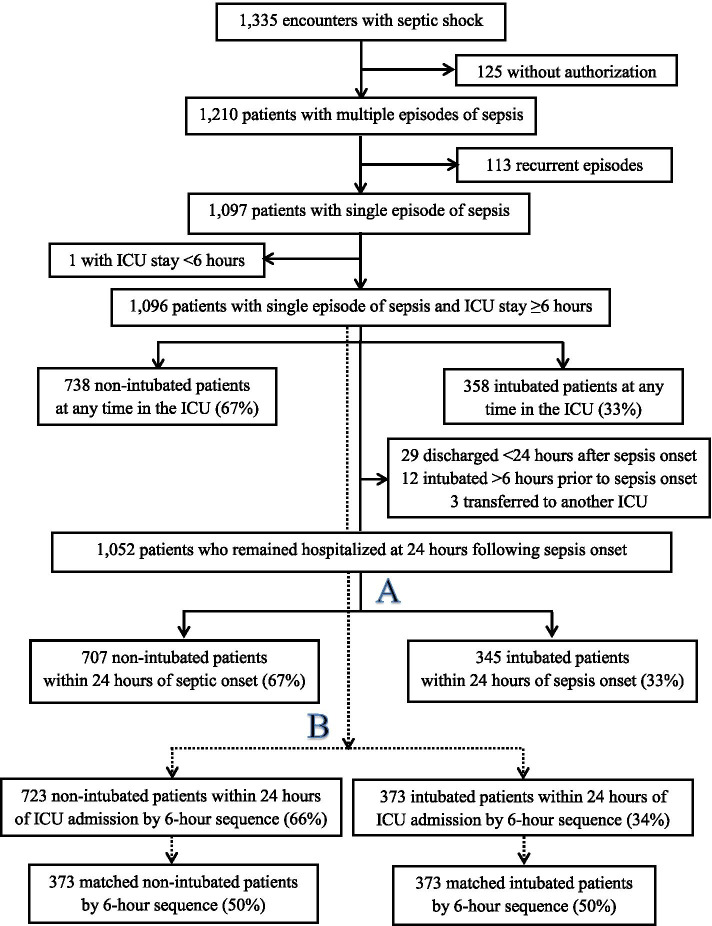
Flowchart: A = intubated in the ICU within 24 h of sepsis onset; B = intubated within 24 h of ICU admission

A total of 1335 encounters were identified between April 1, 2014 and December 31, 2017 of adult patients admitted with septic shock (Fig. 1). Among them, 1096 patients with a single episode of sepsis and ICU stay ≥6 h were eligible, 358 (33%) patients were intubated at any time during their ICU stay and 738 (67%) were not. Overall, the source of infection was clinically suspected in 91% and was microbiologically confirmed in 55% of the cases (Table 1S). The most common sources of infection were lung (32%), abdomen (22%), urinary tract (18%), and skin and soft tissues (12%) with more pulmonary and less abdominal, urinary tract, and skin and soft tissue infections in intubated than in non-intubated patients (*p* < 0.0001) (Table 1S). The main types of infection were Gram-negative bacteria (21%), Gram-positive bacteria (20%), and polymicrobial (11%) with no differences between intubated and non-intubated patients (*p* = 0.579).

**Fig. 2:**
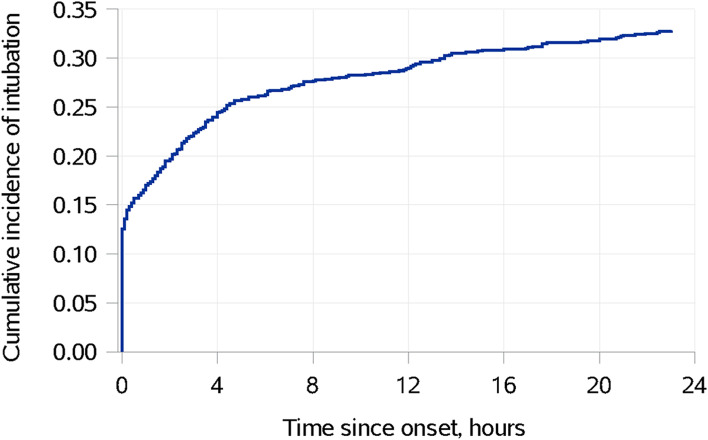
Cumulative incidence of intubation in the 24 h following sepsis diagnosis defined as sepsis onset

After selection and exclusions, 1052 unique patients still in the ICU within 24 h of sepsis onset were further analyzed: 345 (33%) patients were intubated within 24 h and 707 (67%) were not (Table 1) (Fig. 2). Those intubated were younger [median (25th, 75th) percentiles: 66.0 years old (55.4, 74.2) vs. 69.5 (59.4, 80.2), *p* < 0.001], originated more often from an outside facility (45% vs. 35%, *p* = 0.007), had higher median APACHE III score [92 (74, 115) vs. 68 (57, 82), *p* < 0.001] and SOFA score [10 (8,13) vs. 6 (4,8), *p* < 0.001], achieved mean arterial pressure goals within 6 h more often but less often lactate level normalization, and stayed on the ventilator for an average of 2.3 days (1.1, 4.8).

**Table 1:** characteristics of patients who remained hospitalized in the ICU at 24 h following sepsis onset, summarized by intubation requirement

*Variable*	*Non-intubated within 24 h (N = 707)*		*Intubated within 24 h (N = 345)*		*P* Value
Age (y)	69.5	(59.4, 80.2)	66.0	(55.4, 74.2)	< 0.001
Sex					0.24
Male	383	(54)	200	(58)	
Female	324	(32)	145	(33)	
BMI (kg/m^2^), *n* = 701/339	28.2	(23.6, 34.5)	28.5	(24.5, 35.1)	0.18
ICU admission source					0.007
Emergency Department	328	(32)	134	(34)	
Direct admit (from an outside facility)	249	(35)	156	(36)	
Transfer from the floor	130	(18)	55	(16)	
APACHE III score	68	(57, 82)	92	(74, 115)	< 0.001
SOFA score (day 1)	6	(4,8)	10	(8,13)	< 0.001
SOFA score (day 2), *n* = 589/334	4	(2,7)	7	(4,10)	< 0.001
Failed to resolve within 6 h per MAP	113	(16)	30	(9)	0.001
Failed to resolve within 6 h per lactate	230	(37)	137	(38)	0.022
Non-invasive ventilation use	108	(15)	62	(18)	0.26
Days on invasive ventilation, *n* = 153/345	0.8	(0.3, 2.0)	2.3	(1.1, 4.8)	< 0.001
ICU mortality	37	(5)	59	(17)	< 0.001
ICU length of stay (d), *n* = 670/286	2.0	(1.3, 3.1)	3.7	(2.3, 6.9)	< 0.001
Hospital mortality	82	(12)	89	(26)	< 0.001
Hospital length of stay (d), *n* = 625/256	6.8	(4.5, 11.4)	10.3	(6.6, 20.6)	< 0.001

Continuous variables are summarized as median (Q1, Q3) and compared using rank-sum tests. Categorical variables are summarized as n (%) and compared using Chi-squared tests. ICU and hospital length of stay are summarized only for patients who were discharged alive from the ICU and hospital respectively. When information is missing, the number of observations with complete data is presented. Abbreviations: ICU = Intensive Care Unit; APACHE III = Acute Physiology and Chronic Health Evaluation III; SOFA + Sequential Organ Failure Assessment; BMI = Body Mass Index; MAP = Mean Arterial Pressure

### Clinical outcomes: unadjusted

**Fig. 3:**
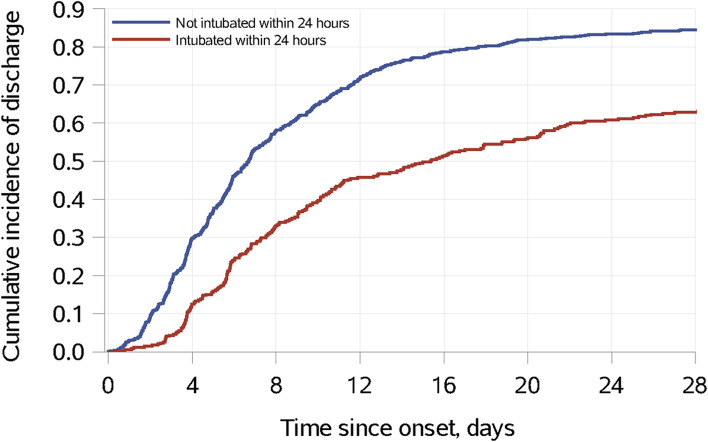
Cumulative incidence of hospital discharge through day 28 in patients alive and in the ICU at 24 h following sepsis diagnosis, defined as sepsis onset, according to intubation status at 24 h following sepsis onset

The crude in-hospital mortality rate was 26% in those intubated within 24 h after sepsis onset and 12% in those not intubated (*p* < 0.001). The crude ICU mortality rate was also higher in the intubated group than the non-intubated group (17% vs. 5%, p < 0.001). The median hospital length of stay, among those discharged alive, was 10.3 days (6.6, 20.6 days) in the intubated group and 6.8 days (4.5, 11.4 days) in the non-intubated group (p < 0.001) (Fig. 3). ICU length of stay was also significantly different between the 2 groups: 3.7 days (2.3, 6.9 days) in the intubated group vs. 2.0 days (1.3, 3.1 days) in the non-intubated group, p < 0.001) (Table 1).

### Clinical outcomes: adjusted

After adjustment for age, sex, ICU admission source, APACHE III and SOFA score on ICU day 1, resolution of hypotension within 6 h, resolution of lactic acidosis within 6 h, and use of non-invasive ventilation, intubation was not associated with hospital mortality [OR 1.00 (95% CI 0.65, 1.55), *p* = 0.99]; however, intubation was associated with decreased hospital-free days through day 28 [estimated difference in hospital-free days − 1.82 (95% CI -3.08, − 0.55), *p* = 0.005] (Table 2).

**Table 2:** Effect of intubation on hospital mortality and hospital-free days in multivariable analysis^a^

*Outcome*	*Estimate (95% CI)*	*P-value*
Hospital mortality	1.00 (0.65, 1.55)	0.999
Hospital-free days	−1.82 (− 3.08, − 0.55)	0.005

^a^Effects of intubation are presented here after adjusting for age, sex, ICU admission source, APACHE III and SOFA score on ICU day 1, resolution of low mean arterial pressure (3 or more consecutive measurements > 65 mmHg) within 6 h, resolution of lactic acidosis (decrease of 50% or normalized) within 6 h, and use of non-invasive ventilation. Hospital mortality was modeled using multivariable logistic regression and estimates are odds ratios where values greater than 1 correspond to an increased likelihood of mortality. Hospital-free days were modeled using multivariable linear regression and negative estimates correspond to a decrease in hospital-free days. Hospital-free days were defined as hospital-free days during the 28 days following sepsis onset with patients who died in the hospital set to 0. Analysis is limited to those patients who were alive 24 h following sepsis onset

### Clinical outcomes: propensity-matched

While there were significant differences between intubated and non-intubated patients in the raw sample, differences in the matched sample were minimal, with absolute standardized differences less than 0.20 (Table 2S). In the propensity-matched sample (Table 3S), there was little evidence that intubation was associated with increased odds of hospital or ICU mortality (OR = 1.23, 95% CI = 0.61, 2.49, *p* = 0.56; and OR = 1.27, 95% CI = 0.57, 2.82, p = 0.56, respectively) (Table 3). Intubation was associated with reduced hospital-free days and ICU-free days through 28 days, with an estimated 3.4 fewer days alive and out of hospital during that time (estimate = − 3.42, 95% CI = -6.11, − 0.74, *p* = 0.013; and estimate = − 2.07, 95% CI = -3.36, − 0.78, *p* = 0.002, respectively).

**Table 3:** Outcomes analysis^a^

	*Estimate (95% CI)*	*P value*
Hospital mortality	1.23 (0.61 to 2.49)	0.562
Hospital-free days	−3.42 (−6.11 to − 0.74)	0.013
ICU mortality	1.27 (0.57 to 2.82)	0.559
ICU-free days	−2.07 (−3.36 to −0.78)	0.002

^a^ For linear and logistic regression, we used generalized estimating equations to account for non-intubated patients selected multiple times as matches. Estimates are odds ratios for mortality endpoints and values above 1 represent increased in odds of event due to early intubation within 24 h of ICU admission. Estimates for length of stay endpoints are for the increase in hospital or ICU-free days associated with early intubation within 24 h of ICU admission (estimates less than 0 indicate longer length of stay and thus, fewer hospital or ICU-free days). To account for uncertainty introduced by multiple imputation, analyses were run separately for each imputation and combined using methods to estimate the between and within sample

## Discussion

In this secondary analysis of a prospectively collected cohort of septic shock patients in a single tertiary center, patients intubated within 24 h after ICU admission were younger and were transferred more often from outside facilities. They presented with higher severity of illness scores, had more lung infections, and more persistent shock. They also had higher ICU and hospital mortality and longer ICU and hospital length of stays. When the analysis was limited to those patients who were alive 24 h following septic shock, and after adjusting for multiple confounders including the use of non-invasive ventilation, intubation was not associated with hospital mortality but was associated with a small decrease in hospital-free days. When the analysis was stratified and matched by time sequence of 6 h within the first 24 h following ICU admission, intubation still was not associated with hospital mortality but still had a small association with hospital-free days at 28 days. These findings suggest that, in patients with septic shock, intubation and invasive mechanical ventilation is not by itself overall a risk factor for increased mortality. This result should help the clinician overcome any hesitation of intubation for fear of worse outcomes, especially in case of acute respiratory distress with predominant hemodynamic compromise, since unnecessarily delaying intubation may worsen outcomes.

Sepsis is a major risk factor for the development of acute hypoxemic respiratory failure especially in the presence of shock [31]. Other factors that contribute to the development of respiratory failure include younger age, higher APACHE II score, a pulmonary source of infection, acute pancreatitis, and acute abdomen [31]. Delayed antibiotics, delayed goal-directed resuscitation, excessive fluid administration and transfusion, lack of source control, and comorbidities (e.g., alcohol dependence, recent chemotherapy) are also contributory [33]. The presence of organ dysfunction defines septic shock and is associated with greater risk of mortality [34]. In sepsis, acute respiratory failure remains associated with worse outcome [7, 35]. Early identification and intervention of patients at risk of acute respiratory failure is possible [36]. In sepsis-related respiratory failure, early liberal and late conservative fluid strategy is associated with better outcomes [37]. Timely intubation may also reduce hospital mortality [13] and prevent further lung injury by limiting contributing factors such as high tidal volumes during spontaneous or non-invasive ventilation [38–40]. In our study, while patients who were intubated and ventilated within 24 h after the onset of septic shock were more critically ill and had higher hospital mortality, intubation itself did not contribute to worse outcomes when adjusted for severity of illness. This raises the possibility that timely intubation when appropriate, coupled with a lung protective strategy, may be well tolerated.

Although some studies suggest that the timing of intubation matters, the data available for patients with sepsis are still limited. Delay in intubation may be associated with worse outcomes [14, 41]. The place of intubation in septic shock may also impact outcome: ICUs with the highest frequency of early intubation (greater than 90% of intubation within 12 h) had a higher mortality rate in comparison to ICUs with middle frequency (between 80 and 90% of early intubation) whereas ICUs with the lowest frequency (less than 80% of patients with early intubation) were associated with increased mortality as well [42]. This finding suggested that some intubations may have been too premature (highest frequency group) or too late (lowest frequency group) and that the timing of intubation itself may impact outcomes. In our study, we did not find the timing of intubation within the first 24 h of septic shock to be a contributing factor for mortality. This outcome may be related to a systematic and structured approach of intubation in our institution with a just-in-time approach to intubation, that is neither too early nor too late [9]. The 2016 updated Surviving Sepsis Campaign guidelines for the management of septic shock only indirectly addresses the role of early intubation by suggesting that noninvasive ventilation should only be used in a minority of sepsis-induced acute respiratory failure patients in whom the benefits outweigh the risks [8]. In our study, the use of non-invasive ventilation was low and similar to what was recently observed in WEAN SAFE, a large multicenter observational study [12]; moreover, the decision to intubate and the timing of intubation were left at the discretion of the care team which did not seem to affect outcome for those who remained alive 24 h after sepsis onset. In our analysis, we also adjusted for the use on non-invasive ventilation as a confounding factor.

This study has several strengths. It encompasses many prospectively and consecutively collected septic shock patients with predetermined standard institutional protocols for intubation and mechanical ventilation as well as sepsis management. Although a difference in outcome was noted in the univariate analysis, both multivariable analysis and propensity score matching using a stratified sampling strategy demonstrated no effect of intubation on hospital mortality. This study has some limitations. First, it is a single center study and the results may not be generalizable. Second, in the primary analysis the cohort was defined by ICU admitted patients, where sepsis onset and possibly intubation could occur shortly after ICU admission (less than 4% had sepsis onset > 6 h prior to ICU admission). To reduce potential for immortal time bias should this interval differ between groups, we used a landmark analysis at + 24 h from sepsis onset (eligibility period) so that the baseline timepoint, sepsis onset, is after the cohort has been defined by ICU admission and diagnosis of sepsis (time of cohort entry) [43]. In the second analysis, we matched patients intubated after admission and within 24 h of admission to patients admitted to the ICU but not intubated at a similar timepoint which reduces potential selection bias. While these two methods implement robust approaches to reduce potential biases, we are unable to fully exclude the possibility of residual bias due to these underlying causes. The source of infection was not always confirmed, which is common in sepsis. Moreover, other causes of shock (e.g. cardiogenic or hemorrhagic) were excluded from the registry with reasonable clinical accuracy. Third, many variables go into a decision to intubate which are not well-collected in the electronic health record and we were unable to account for these: indication for intubation, care limitation (e.g. do-no-intubate and do-not-resuscitate outside the initial 48 h which were an exclusion criteria) [44], decision to intubate, choice of the induction drug(s) used for anesthesia [45], immediate complications after intubation, ventilator setting, and compliance with the sepsis bundle. Fourth, in one of the two analyses, we limited the cohort to patients who were still hospitalized 24 h after ICU admission for septic shock. Fifth, whether some patients were immunocompromised was not specified. Sixth, this was a secondary analysis, and the possibility of unmeasured confounding factors remains [46]. However, to limit the risk of confounding, we performed two sets of analysis, regression modeling and propensity scoring, both showing that even if patients who required intubation had higher severity score and higher crude mortality, intubation itself within the first 24 h following ICU admission did not influence outcome as expressed as hospital-free days. Finally, some variables identified as potential confounders were defined in the time-period simultaneous to our exposure (intubation) and in the linear regression model may have been ascertained after exposure but before observation of the outcome. Our second approach using propensity score matching provides robustness to this by only using confounders ascertained prior to intubation.

## Conclusions

Intubation and invasive mechanical ventilation that occurred within 24 h after ICU admission in adult patients with septic shock was not associated with hospital mortality but was associated with reduced 28-day hospital-free days. Although intubation remains a high-risk procedure in critically ill adults, our study did not identify an increased risk in mortality among patients with septic shock who exhibited hemodynamic compromise.

## Supplementary Information

**Additional file 1.**

## Data Availability

The datasets generated and/or analyzed during the current study are not publicly available due Institution Data Sharing Agreement Policy but are available from the corresponding author on reasonable request. This work was presented at ESICM LIVES 2018, 31st Annual Congress in Paris, France.

## Abbreviations

APACHE-III: Acute Physiology and Chronic Health Evaluation-III score
APS: Acute Physiology Score
BUN: Blood Urea Nitrogen
ICU: Intensive Care Unit
MAP: Mean Arterial Pressure
PBW: Predicted Body Weight
SOFA: Sequential Organ Failure Assessment score

## References

[1] Kadri SS, Rhee C, Strich JR (2017). Estimating ten-year trends in septic shock incidence and mortality in United States academic medical centers using clinical data. Chest..

[2] Rhee C, Dantes R (2017). Epstein L, et al; CDC prevention epicenter program: incidence and trends of Sepsis in US hospitals using clinical vs claims data, 2009-2014. JAMA..

[3] Driessen RGH, van de Poll MCG, Mol MF, van Mook WNKA, Schnabel RM (2018). The influence of a change in septic shock definitions on intensive care epidemiology and outcome: comparison of sepsis-2 and sepsis-3 definitions. Infect Dis (Lond).

[4] Weisberg A, Park P, Cherry-Bukowiec JR (2018). Early goal-directed therapy: the history and ongoing impact on Management of Severe Sepsis and Septic Shock. Surg Infect.

[5] Levy MM, Evans LE, Rhodes A (2018). The surviving Sepsis campaign bundle: 2018 update. Crit Care Med.

[6] Mikkelsen ME, Shah CV, Meyer NJ (2013). The epidemiology of acute respiratory distress syndrome in patients presenting to the emergency department with severe sepsis. Shock..

[7] Auriemma CL, Zhuo H, Delucchi K, Deiss T, Liu T, Jauregui A, Ke S, Vessel K, Lippi M, Seeley E, Kangelaris KN, Gomez A, Hendrickson C, Liu KD, Matthay MA, Ware LB, Calfee CS (2020). Acute respiratory distress syndrome-attributable mortality in critically ill patients with sepsis. Intensive Care Med.

[8] Rhodes A, Evans LE, Alhazzani W (2017). Surviving Sepsis campaign: international guidelines for Management of Sepsis and Septic Shock: 2016. Crit Care Med.

[9] Bauer PR, Kumbamu A, Wilson ME (2017). Timing of intubation in acute respiratory failure associated with Sepsis: a mixed methods study. Mayo Clin Proc.

[10] Darreau C, Martino F, Saint-Martin M (2020). Use, timing and factors associated with tracheal intubation in septic shock: a prospective multicentric observational study. Ann Intensive Care.

[11] Kangelaris KN, Ware LB, Wang CY (2016). Timing of intubation and clinical outcomes in adults with acute respiratory distress syndrome. Crit Care Med.

[12] Bellani G, Laffey JG, Pham T (2017). Noninvasive ventilation of patients with acute respiratory distress syndrome. Insights from the LUNG SAFE study. Am J Respir Crit Care Med.

[13] Bauer PR, Gajic O, Nanchal R (2017). Association between timing of intubation and outcome in critically ill patients: a secondary analysis of the ICON audit. J Crit Care.

[14] Hraiech S, Alingrin J, Dizier S (2013). Time to intubation is associated with outcome in patients with community-acquired pneumonia. PLoS One.

[15] Perbet S, De Jong A, Delmas J (2015). Incidence of and risk factors for severe cardiovascular collapse after endotracheal intubation in the ICU: a multicenter observational study. Crit Care.

[16] Demoule A, Hill N, Navalesi P (2016). Can we prevent intubation in patients with ARDS?. Intensive Care Med.

[17] Simpson GD, Ross MJ, McKeown DW, Ray DC (2012). Tracheal intubation in the critically ill: a multi-Centre national study of practice and complications. Br J Anaesth.

[18] Russotto V, Myatra SN, Laffey JG (2021). Intubation practices and adverse Peri-intubation events in critically ill patients from 29 countries. JAMA..

[19] de Montmollin E, Aboab J, Ferrer R, Azoulay E, Annane D (2016). Criteria for initiation of invasive ventilation in septic shock: an international survey. J Crit Care.

[20] Stolbach AI, Hoffman RS, Nelson LS (2008). Mechanical ventilation was associated with acidemia in a case series of salicylate-poisoned patients. Acad Emerg Med.

[21] The Strengthening the Reporting of Observational Studies in Epidemiology (STROBE) Statement: guidelines for reporting observational studies. https://www.equator-network.org/reporting-guidelines/strobe/. Accessed 12 Dec 2020. 10.1136/bmj.39335.541782.AD PMC2034723 17947786

[22] Dellinger RP, Levy MM, Rhodes A (2013). Surviving sepsis campaign: international guidelines for management of severe sepsis and septic shock: 2012. Crit Care Med.

[23] Siontis B, Elmer J, Dannielson R (2015). Multifaceted interventions to decrease mortality in patients with severe sepsis/septic shock-a quality improvement project. PeerJ..

[24] Harrison AM, Thongprayoon C, Kashyap R (2015). Developing the surveillance algorithm for detection of failure to recognize and treat severe sepsis. Mayo Clin Proc.

[25] Herasevich V, Kor DJ, Li M, Pickering BW: ICU data mart: a non-iT approach. A team of clinicians, researchers and informatics personnel at the Mayo Clinic have taken a homegrown approach to building an ICU data mart. Healthc Inform*.* 2011;28(11):42, 44–45. 22121570

[26] Austin PC, Fine JP (2017). Practical recommendations for reporting Fine-gray model analyses for competing risk data. Stat Med.

[27] Allison P. For causal analysis of competing risks, Don’t Use Fine & Gray’s Subdistribution Method https://statisticalhorizons.com/for-causal-analysis-of-competing-risks. Accessed 03 June 2021.

[28] Lesko CR, Lau B (2017). Bias due to confounders for the exposure-competing risk relationship. Epidemiology..

[29] Yehya N, Harhay MO, Curley MAQ, Schoenfeld DA, Reeder RW (2019). Reappraisal of ventilator-free days in critical care research. Am J Respir Crit Care Med.

[30] Harhay MO, Ratcliffe SJ, Small DS, Suttner LH, Crowther MJ, Halpern SD (2019). Measuring and analyzing length of stay in critical care trials. Med Care.

[31] Seethala RR, Hou PC, Aisiku IP (2017). Early risk factors and the role of fluid administration in developing acute respiratory distress syndrome in septic patients. Ann Intensive Care.

[32] van Buuren S (2007). Multiple imputation of discrete and continuous data by fully conditional specification. Stat Methods Med Res.

[33] Kyriacou DN, Lewis RJ (2016). Confounding by indication in clinical research. JAMA..

[34] Delbove A, Darreau C, Hamel JF, Asfar P, Lerolle N (2015). Impact of endotracheal intubation on septic shock outcome: a post hoc analysis of the SEPSISPAM trial. J Crit Care.

[35] Frat JP, Ragot S, Coudroy R (2018). Predictors of intubation in patients with acute hypoxemic respiratory failure treated with a noninvasive oxygenation strategy. Crit Care Med.

[36] Law AC, Stevens JP, Walkey AJ (2019). National Trends in timing of death among patients with septic shock. 1994-2014. Crit Care Med.

[37] Wan C, Hanson AC, Schulte PJ, Dong Y, Bauer PR (2021). Propofol, ketamine, and Etomidate as induction agents for intubation and outcomes in critically ill patients: a retrospective cohort study. Crit Care Explor.

[38] Iscimen R, Cartin-Ceba R, Yilmaz M (2008). Risk factors for the development of acute lung injury in patients with septic shock: an observational cohort study. Crit Care Med.

[39] Grieco DL, Menga LS, Eleuteri D, Antonelli M (2019). Patient self-inflicted lung injury: implications for acute hypoxemic respiratory failure and ARDS patients on non-invasive support. Minerva Anestesiol.

[40] Singer M, Deutschman CS, Seymour CW (2016). The third international consensus definitions for Sepsis and septic shock (Sepsis-3). JAMA..

[41] Gajic O, Dabbagh O, Park PK (2011). Early identification of patients at risk of acute lung injury: evaluation of lung injury prediction score in a multicenter cohort study. Am J Respir Crit Care Med.

[42] Murphy CV, Schramm GE, Doherty JA (2009). The importance of fluid management in acute lung injury secondary to septic shock. Chest..

[43] Carteaux G, Millán-Guilarte T, De Prost N (2016). Failure of noninvasive ventilation for De novo acute hypoxemic respiratory failure: role of tidal volume. Crit Care Med.

[44] Thille AW, Contou D, Fragnoli C (2013). Non-invasive ventilation for acute hypoxemic respiratory failure: intubation rate and risk factors. Crit Care.

[45] Vail EA, Gershengorn HB, Wunsch H, Walkey AJ (2021). Attention to immortal time Bias in critical care research. Am J Respir Crit Care Med.

[46] Mehter HM, Wiener RS, Walkey AJ (2014). "do not resuscitate" decisions in acute respiratory distress syndrome. A secondary analysis of clinical trial data. Ann Am Thorac Soc.

