# Supplementary material for: Outcome after intubation for septic shock with respiratory distress and hemodynamic compromise: an observational study
Source: BMC Anesthesiol. 2021 Oct 25;21:253. doi: 10.1186/s12871-021-01471-x (PMC8543776; doi:10.1186/s12871-021-01471-x)
Supplement: Supplementary file 1 — Additional file 1. [file 12871_2021_1471_MOESM1_ESM.docx]

**Outcome After Intubation For Septic Shock With Respiratory Distress And Hemodynamic Compromise: An Observational Study**Ting Yang MD^1,2^,Yongchun Shen, MD^1,2^, John G. Park, MD^2^, Phillip J. Schulte PhD^3^, Andrew C. Hanson MS^3^, Vitaly Herasevich MD^4^, Yue Dong MD^4^, Philippe R. Bauer, MD, PhD^2^

1. Respiratory and Critical Care Medicine, West China Hospital, Sichuan University, Chengdu, Sichuan 610041, China

2. Pulmonary and Critical Care Medicine, Mayo Clinic, Rochester, Minnesota 55905, USA

3. Health Science Research – Biomedical Statistics and Informatics, Mayo Clinic, Rochester, Minnesota 55905, USA

4. Critical Care Medicine, Mayo Clinic, Rochester, Minnesota 55905, USA

**Corresponding author**
Philippe R. Bauer M.D., Ph.D.
Pulmonary and Critical Care Medicine
Mayo Clinic, Rochester, MN 55905, USA
Phone: 507-266-3958
E-mail: [Bauer.Philippe@mayo.edu](mailto:Bauer.Philippe@mayo.edu)

**Table 1S**: Source and type of infection among patients with single episode of sepsis and ICU stay ≥6 hours summarized by intubation requirement

| *Variable* | *Total (N=1,096)* | | *Non-intubated anytime (N=738)* | | *Intubated anytime (N=358)* | | *P* Value |
| --- | --- | --- | --- | --- | --- | --- | --- |
| Source of infection |  |  |  |  |  |  | <0.0001 |
| Urinary tract | 195 | (18) | 157 | (21) | 38 | (11) | <0.0001 |
| Lung | 351 | (32) | 165 | (22) | 186 | (52) | <0.0001 |
| Abdomen | 242 | (22) | 183 | (25) | 59 | (16) | 0.0019 |
| Skin and soft tissue | 129 | (12) | 100 | (14) | 29 | (8) | 0.0086 |
| Line and device | 26 | (2) | 25 | (3) | 1 | (<1) | 0.0015 |
| Heart | 17 | (2) | 11 | (2) | 6 | (2) | 0.8157 |
| Joint and bone | 16 | (1) | 11 | (2) | 5 | (1) | 0.9032 |
| Nose and throat | 12 | (1) | 9 | (1) | 3 | (1) | 0.5692 |
| Neutropenia | 8 | (1) | 7 | (1) | 1 | (<1) | - |
| Central nervous system | 3 | <1) | 0 | (0) | 3 | (1) | - |
| Unknown | 97 | (9) | 70 | (9) | 27 | (8) | 0.2881 |
| Type of infection |  |  |  |  |  |  | 0.5790 |
| Gram negative bacteria | 226 | (21) | 162 | (22) | 64 | (18) | - |
| Gram positive bacteria | 217 | (20) | 151 | (20) | 66 | (18) | - |
| Polymicrobial | 118 | (11) | 80 | (11) | 38 | (11) | - |
| Viruses | 22 | (2) | 13 | (2) | 9 | (3) | - |
| Fungi | 11 | (1) | 6 | (1) | 5 | (1) | - |
| Mycobacteria | 2 | (<1) | 1 | (<1) | 1 | (<1) | - |
| Parasites | 2 | (<1) | 1 | (<1) | 1 | (<1) | - |
| Unknown | 498 | (45) | 324 | (44) | 174 | (49) | - |

Categorical variables are summarized as n (%) and compared using Chi-squared tests. Only the number of observations with complete data is presented.

**Table 2S**: Patient characteristics for the full and matched cohorts

|  | *Full cohort* | | | | | *Matched cohort†* | | | | |
| --- | --- | --- | --- | --- | --- | --- | --- | --- | --- | --- |
|  | *Non- intubated within 24 hours (n=723)* | | *Intubated within 24 hours (n=373)* | | *Absolute  Std. Diff.* | *Non- intubated (n=373)* | | *Intubated (n=373)* | | *Absolute  Std. Diff.* |
| Age (y) | 69 | (59, 80) | 66 | (55, 74) | 0.28 | 65 | (56, 75) | 66 | (55, 74) | 0.04 |
| Sex |  |  |  |  | 0.10 |  |  |  |  | 0.19 |
| Male | 391 | (54) | 220 | (59) |  | 185 | (50) | 220 | (59) |  |
| Female | 332 | (46) | 153 | (41) |  | 188 | (50) | 153 | (41) |  |
| BMI (kg/m^2^), n=717/366 | 28 | (24, 34) | 29 | (25, 35) | 0.10 | 30 | (26, 41) | 29 | (25, 35) | 0.25 |
| ICU admission source |  |  |  |  |  |  |  |  |  |  |
| Emergency department | 336 | (46) | 146 | (39) | 0.15 | 168 | (45) | 146 | (39) | 0.12 |
| Direct admit | 255 | (35) | 168 | (45) | 0.20 | 146 | (39) | 168 | (45) | 0.12 |
| Transfer from the floor | 132 | (18) | 59 | (16) | 0.06 | 59 | (16) | 59 | (16) | 0.00 |
| APS, n=721/373§ | 54 | (43, 65) | 80 | (64, 102) | 1.20 | 48 | (26, 58) | 43 | (24, 61) | 0.04 |
| SOFA score§ | 6 | (4, 8) | 11 | (8, 13) | 1.24 | 5 | (2, 7) | 4 | (2, 7) | 0.03 |
| \| Intubated at sepsis onset \| \| --- \| |  | NA | 148 | (40) | - |  | NA | 148 | (40) | - |
| Failed to resolve within 6 hours per MAP | 116 | (16) | 35 | (9) | 0.20 | 65 | (17) | 35 | (9) | 0.24 |
| Failed to resolve within 6 hours per lactate | 243 | (34) | 152 | (41) | 0.15 | 179 | (48) | 152 | (41) | 0.15 |

Categorical variables are presented as n (%) and continuous variables are presented as median (Q1, Q3). When not all data are present, the numbers with available data are presented by treatment category.
† Multiple imputations were used to impute missing data. In this summary, data from a single imputation is presented.
§ In the full cohort summary, day 1 APS and SOFA scores are presented. Because patients were selected into the study at various time-points, APS and SOFA scores calculated from data available within the 6-hour time-frame prior to selection into the study are reported for the matched cohort.
Abbreviations: Std. Diff. = standardized difference; y = years; kg = kilograms; m = meters; ICU = Intensive Care Unit; APS = Acute Physiology Score; SOFA = Sequential Organ Failure Assessment; MAP = mean arterial pressure.

**Table 3S**: Variables included in the propensity score calculation*

|  | *Not intubated (N=373)* | | *Intubated (N=373)* | | *Absolute Std. Diff.* |
| --- | --- | --- | --- | --- | --- |
| Patient characteristics |  |  |  |  |  |
| Age (y) | 65 | (56, 75) | 66 | (55, 74) | 0.04 |
| Sex |  |  |  |  | 0.19 |
| Male | 185 | (50) | 220 | (59) |  |
| Female | 188 | (50) | 153 | (41) |  |
| ICU admission source |  |  |  |  |  |
| ED | 168 | (45) | 146 | (39) | 0.12 |
| Direct admit from | 146 | (39) | 168 | (45) | 0.12 |
| Transfer from floor | 59 | (16) | 59 | (16) | 0.00 |
| Pre-ICU hospital length of stay |  |  |  |  |  |
| < 2 hours | 317 | (85) | 313 | (84) | 0.03 |
| 2-23.9 hours | 40 | (11) | 29 | (8) | 0.10 |
| ≥ 24 hours | 16 | (4) | 31 | (8) | 0.17 |
| Acute physiology score§ | 48 | (26, 58) | 43 | (24, 61) | 0.04 |
| Admission year | 2016 | (2014, 2017) | 2016 | (2014, 2017) | 0.01 |
| Laboratory values† |  |  |  |  |  |
| Anion gap (mEq/L; n=259/232) | 16 | (14, 20) | 16 | (13, 20) | 0.03 |
| Bicarbonate (mEq/L; n=289/294) | 21 | (17, 26) | 21 | (16, 25) | 0.05 |
| Hematocrit (percent; n=280/260) | 33 | (28, 39) | 34 | (28, 40) | 0.05 |
| Potassium (mEq/L; n=280/276) | 4.4 | (3.9, 4.9) | 4.4 | (3.8, 4.9) | 0.10 |
| Creatinine (mg/dL; n=265/244) | 1.7 | (1.0, 2.8) | 1.5 | (1.0, 2.4) | 0.03 |
| Glucose (mg/dL; n=303/292) | 134 | (105, 204) | 134 | (100, 185) | 0.07 |
| Sodium (mEq/L; n=275/274) | 136 | (132, 140) | 137 | (133, 141) | 0.05 |
| BUN (mg/dL; n=237/195) | 27 | (16, 46) | 27 | (16, 45) | <0.01 |
| Bilirubin (mg/dL; n=161/118) | 0.8 | (0.5, 3.3) | 1.1 | (0.5, 4.9) | 0.06 |
| Lactate (mmol/L; n=284/265) | 3.0 | (1.8, 4.5) | 2.8 | (1.6, 4.7) | 0.08 |
| pH (n=194/209) | 7.3 | (7.2, 7.3) | 7.3 | (7.2, 7.4) | 0.02 |

* Values are n (%) for categorical variables and median (Q1, Q3) for continuous variables except for admission year which is summarized as median (minimum, maximum). Missing values have been imputed using multiple imputation and values from a single imputation are summarized. Where values have been imputed, the numbers of patients with non-missing data prior to imputation are summarized. § Acute physiology score (APS) is based on available data over the 6 hour period prior to the study. Missing laboratory values added no points to the total score and due to this method, reported APS is likely an underestimate of true severity of illness.† Laboratory values were obtained from the 6 hour period prior to selection into the study. When no value was available over that period, values were imputed using multiple imputations. Abbreviations: BUN = Blood Urea Nitrogen.
